# Evaluation of the orally bioavailable 4-phenylbutyrate-tethered trichostatin A analogue AR42 in models of spinal muscular atrophy

**DOI:** 10.1038/s41598-023-37496-0

**Published:** 2023-06-26

**Authors:** Casey J. Lumpkin, Ashlee W. Harris, Andrew J. Connell, Ryan W. Kirk, Joshua A. Whiting, Luciano Saieva, Livio Pellizzoni, Arthur H. M. Burghes, Matthew E. R. Butchbach

**Affiliations:** 1 Division of Neurology, Nemours Children’s Hospital Delaware, 4462 E400 DuPont Experimental Station, 200 Powder Mill Road, Wilmington, DE 19803 USA; 2 Department of Biological Sciences, University of Delaware, Newark, DE USA; 3 Department of Pathology and Cell Biology, Columbia University, New York, NY USA; 4 Department of Neurology, Columbia University, New York, NY USA; 5 Center for Motor Neuron Biology and Disease, Columbia University, New York, NY USA; 6 Department of Biological Chemistry and Pharmacology, The Ohio State University Wexner Medical Center, Columbus, OH USA; 7 Department of Neurology, The Ohio State University Wexner Medical Center, Columbus, OH USA; 8 Department of Pediatrics, Thomas Jefferson University, Philadelphia, PA USA

**Keywords:** Drug discovery, Genetics, Neuroscience

## Abstract

Proximal spinal muscular atrophy (SMA) is a leading genetic cause for infant death in the world and results from the selective loss of motor neurons in the spinal cord. SMA is a consequence of low levels of SMN protein and small molecules that can increase SMN expression are of considerable interest as potential therapeutics. Previous studies have shown that both 4-phenylbutyrate (4PBA) and trichostatin A (TSA) increase SMN expression in dermal fibroblasts derived from SMA patients. AR42 is a 4PBA-tethered TSA derivative that is a very potent histone deacetylase inhibitor. SMA patient fibroblasts were treated with either AR42, AR19 (a related analogue), 4PBA, TSA or vehicle for 5 days and then immunostained for SMN localization. AR42 as well as 4PBA and TSA increased the number of SMN-positive nuclear gems in a dose-dependent manner while AR19 did not show marked changes in gem numbers. While gem number was increased in AR42-treated SMA fibroblasts, there were no significant changes in *FL-SMN* mRNA or SMN protein. The neuroprotective effect of this compound was then assessed in SMNΔ7 SMA (*SMN2*^+*/*+^*;SMNΔ7*^+*/*+^*;mSmn*^*−/−*^) mice. Oral administration of AR42 prior to disease onset increased the average lifespan of SMNΔ7 SMA mice by ~ 27% (20.1 ± 1.6 days for AR42-treated mice vs. 15.8 ± 0.4 days for vehicle-treated mice). AR42 treatment also improved motor function in these mice. AR42 treatment inhibited histone deacetylase (HDAC) activity in treated spinal cord although it did not affect SMN protein expression in these mice. AKT and GSK3β phosphorylation were both significantly increased in SMNΔ7 SMA mouse spinal cords. In conclusion, presymptomatic administration of the HDAC inhibitor AR42 ameliorates the disease phenotype in SMNΔ7 SMA mice in a SMN-independent manner possibly by increasing AKT neuroprotective signaling.

## Introduction

Proximal spinal muscular atrophy (SMA) is an autosomal recessive, early-onset neurodegenerative disease characterized by selective loss of α motor neurons in the anterior horn of the spinal cord^1,2^.This loss of α motor neurons leads to atrophy of limb and trunk muscles. SMA results from the loss or mutation of the *SMN1* (*survival motor neuron*) gene^3^. In humans, *SMN1* is duplicated and the two genes, *SMN1* and *SMN2*, functionally differ by a single cytosine to thymine transition within exon 7^4,5^. While translation of transcripts from *SMN1* produce full-length SMN (FL-SMN) protein, most of the transcripts from *SMN2* lack exon 7 (SMNΔ7) and produce a truncated, unstable SMNΔ7 protein. About 10–20% of *SMN2* transcripts are alternatively spliced to include exon 7 which then results in the production of FL-SMN protein^4,5^. Although SMA results from the loss of a single gene, it has a wide clinical spectrum of disease severity^1^. The severity of SMA depends on the copy number of *SMN2* and the consequent levels of the SMN protein (reviewed in^6^). *SMN2* is, therefore, a genetic modifier of disease severity in SMA.

Unlike humans, mice carry only one *SMN* gene (*mSmn* or *Smn1*) which is orthologous to *SMN1*^7,8^. Loss of *mSmn* results in embryonic lethality in the mouse suggesting that the *mSmn* gene product is essential for cell function and survival^9^. Transgenic insertion of *SMN2* into *mSmn* null mice rescues the embryonic lethality phenotype^10,11^. However, mice with low copy numbers (i.e. 1–2) of *SMN2* develop severe SMA and die at 6–8 days^10–12^. Increasing *SMN2* copy number results in a milder phenotype in the SMA mice^12^. When the *SMN2* copy number is high, the resultant SMA mice are phenotypically indistinguishable from non-SMA littermates^11^. Introducing SMN lacking exon 7 (SMNΔ7)—the predominant mRNA produced by *SMN2*—into a severe SMA mouse genetic background partially ameliorates the SMA phenotype and these mice die at 14–15 days^13^. This suggests that SMNΔ7 might be partially functional but not enough to completely rescue SMA-like motor neuron degeneration. Collectively, these studies demonstrate that increasing *SMN2* expression ameliorates the SMA phenotype in mouse model as it does in humans, making *SMN2* a desirable target for the development of therapeutic agents. Accordingly, there are currently three different FDA-approved SMN-inducing therapies for SMA which either modulate *SMN2* splicing or aim to replace *SMN1*: nusinersen (Spinraza)^14,15^, risdiplam (Evrysdi)^16^ and onasemnogene abeparvovec (Zolgensma)^17^.

*SMN2* expression can be increased by inhibiting histone deacetylase (HDAC) activity which remodels the chromatin containing this gene^18^. Many different HDAC inhibitors of different classes increase *SMN2* expression in cultured cells. These *SMN2*-inducing HDAC inhibitors include butyric acid (BA;^19^), 4-phenylbutyric acid (4PBA;^20,21^), valproic acid (VPA;^22–25^), trichostatin A (TSA;^26^), suberoylanilide hydroxamic acid (SAHA;^27–29^), M344^27,29^, MS-275^27^, *m*-carboxycinnamic acid bis-hydroxamide (CBHA;^27^), LBH589^30^ and JNJ-26481585^31^. It is thought that HDAC inhibitors increase *SMN2* promoter activity as a result of increased acetylation of histones^32^. Accordingly, using isoform-specific short hairpin RNAs, knockdown of all HDAC isoforms, except for HDAC5, increases *SMN2* promoter activity^33^.

TSA improves the survival and phenotype of SMNΔ7 SMA mice following repeated intraperitoneal injections^26,34,35^. We have previously demonstrated that short-chain fatty acid derivative 4PBA significantly improves the survival of SMNΔ7 SMA mice when administered orally^36^. AR42 is a novel, orally bioavailable HDAC inhibitor that is 4PBA-tethered derivative of TSA^37,38^. As TSA and 4PBA both increase *SMN2* expression in SMA cells and exert protective effects on mouse models for SMA, we examined the effect of AR42 on the expression of *SMN2* in human SMA fibroblasts as well on the survival and phenotype of SMNΔ7 SMA mice.

## Results

### Effects of AR42 and AR19 on SMN expression in cultured cells

**Figure 1:**
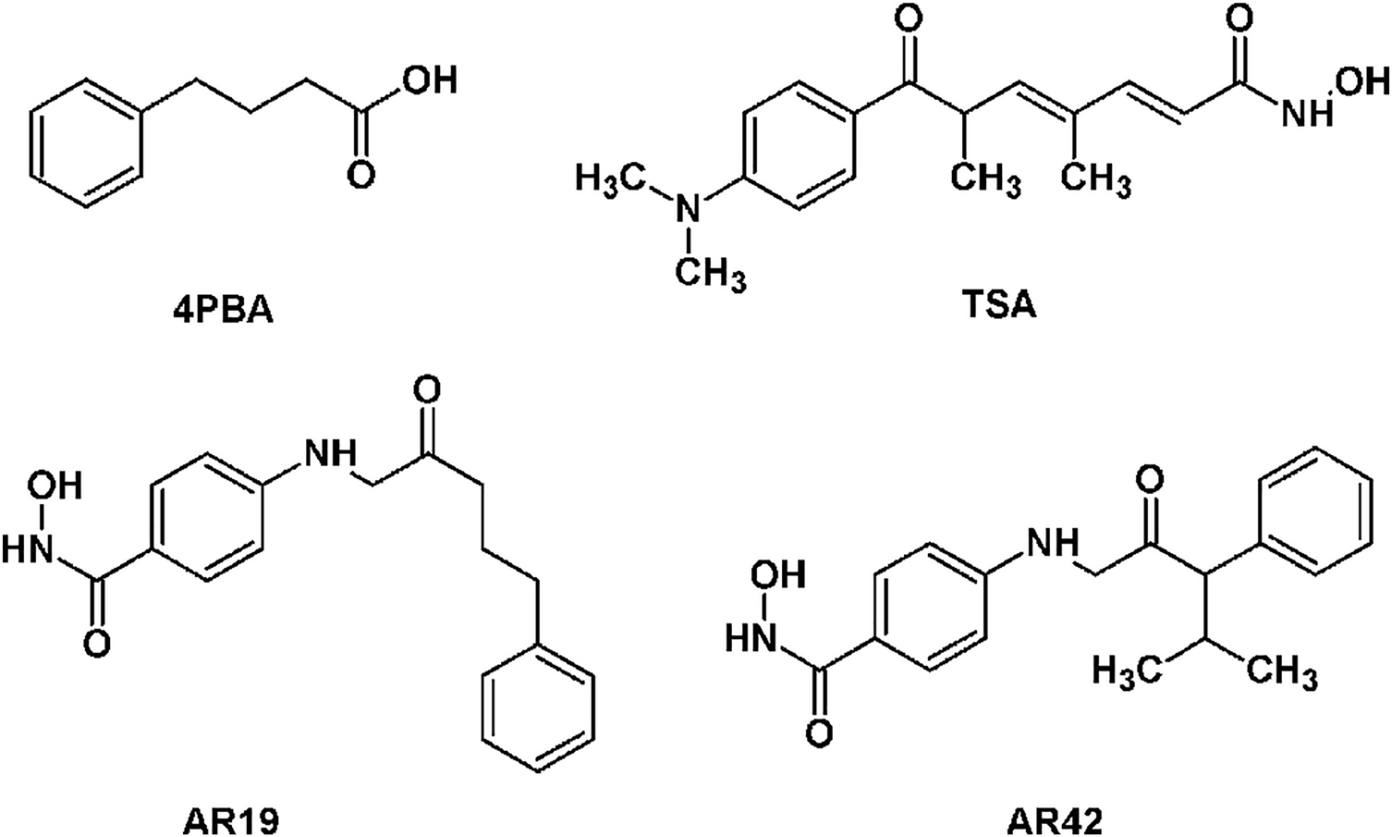
Chemical structures of the compounds tested.

**Figure 2:**
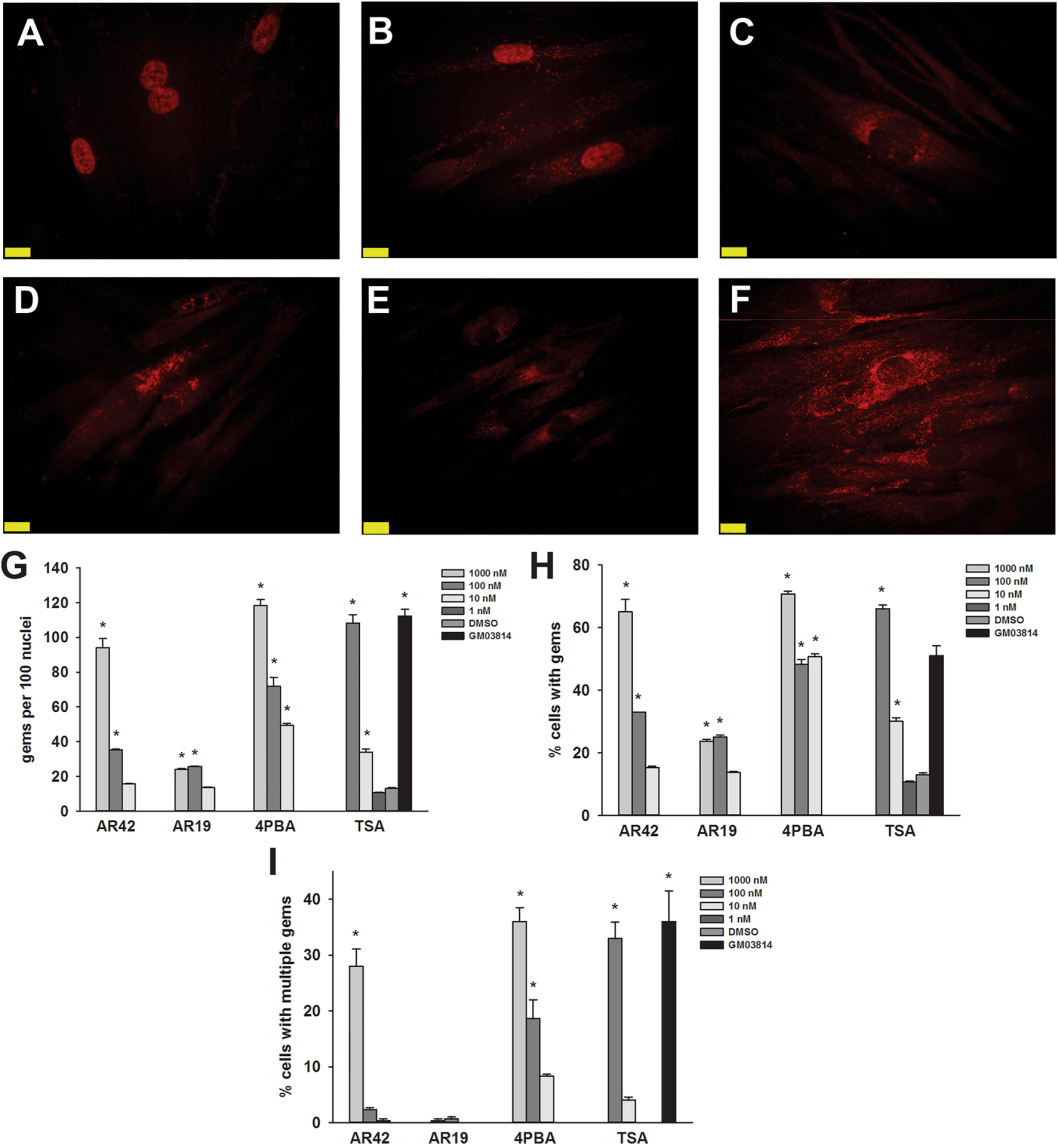
Effect of AR42 on gem numbers in type II SMA fibroblasts. GM03813 type II SMA fibroblasts were treated with differing doses of AR42, AR19, 4PBA or TSA for 5 days (n = 3/dose/drug). The number of SMN-positive gems within 100 randomly selected nuclei was counted. Representative images are shown for GM03813 SMA fibroblasts treated with 1 μM AR42 (**A**), 1 μM AR19 (**B**), 1 μM 4PBA (**C**), 100 nM TSA (**D**) or DMSO (**E**) as well as for GM03814 carrier fibroblasts (**F**). The scale bars (yellow lines) represent 10 μm. The gem count analysis was expressed as (**G**) the number of gems per 100 nuclei, (**H**) the proportion of cells containing gems and (**I**) the proportion of cells containing multiple gems. The data are presented as mean ± standard error. The asterisk (*) denotes a statistically significant (p ≤ 0.05; one-way ANOVA with Bonferroni post hoc test) difference between drug- and vehicle-treated GM03813 SMA cells.

SMN localizes to the cytosol and discreet nuclear bodies called gems^39^. Subnuclear gems can be detected in cell using immunohistochemistry. The number of nuclei with gems and the number of gems per cell is reduced in SMA fibroblasts^40^. As TSA and 4PBA increase the number of gems in SMA fibroblasts^20,21,26^, we examined the effect of AR42 and its less potent analogue AR19 (Fig. 1) on SMN localization to gems in GM03813 type II SMA fibroblasts. AR42 (Fig. 2A) along with TSA (Fig. 2C) and 4PBA (Fig. 2D) markedly increased the number of SMN-positive gems in GM03813 fibroblasts in a dose-dependent manner relative to DMSO-treated GM03813 fibroblasts (Fig. 2E). AR19, on the other hand, had a marginal effect on subnuclear localization of SMN in these cells (Fig. 2B). Treatment of GM03813 cells with 1 μM TSA for 5 days resulted in marked toxicity (as assessed by SMN immunohistochemistry; data not shown); the other compounds tested did not show any toxicity at this dose. AR42, TSA and 4PBA increased the number of gems (Fig. 2G), the proportion of SMA cells containing gems (Fig. 2H) as well as the proportion of SMA cells containing multiple gems (Fig. 2I) in a dose-dependent manner. In fact, the number of gems approaches the number seen in carrier fibroblasts (GM03814; Fig. 2F) at the highest doses tested.

**Figure 3:**
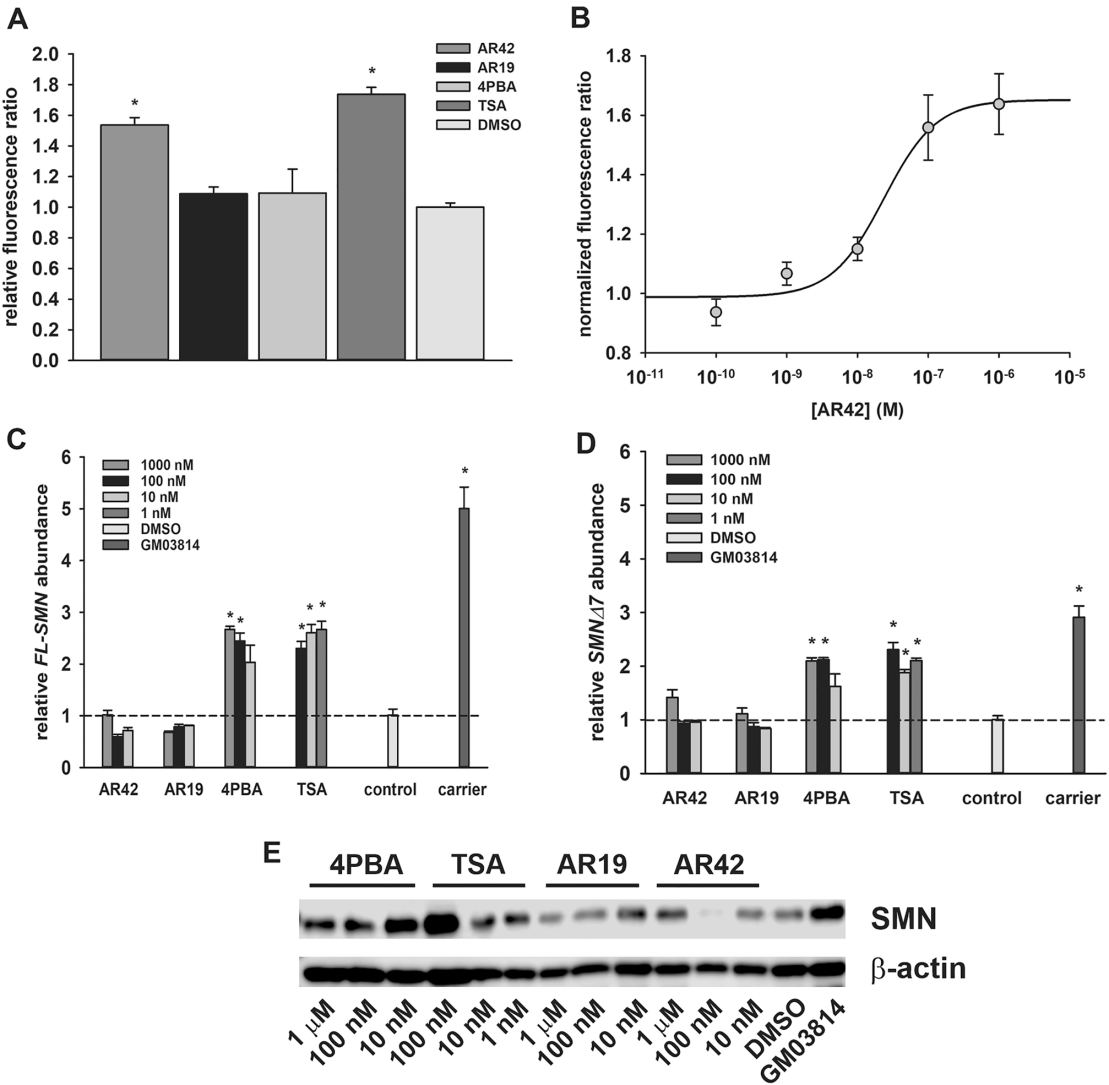
Effects of AR42 on SMN expression in cultured cells. (**A**) *SMN2* promoter activity, expressed as the fluorescence ratio (F460:F530) relative to that for DMSO, in clone 11 NSC34 cells treated with 100 nM AR42, AR19, 4PBA or TSA for 19 h. (**B**) *SMN2* promoter dose–response curve for AR42 treatment. (**C**) Changes in *SMN-FL* mRNA levels in GM03813 SMA fibroblasts treated with different doses of AR42, AR19, 4PBA and TSA for 5 days. The *SMN-FL* mRNA level in GM03814 healthy carrier fibroblasts was also measured. The *SMN-FL* mRNA levels were normalized to those of DMSO-treated GM03813 SMA fibroblasts (dashed line). (**D**) Changes in *SMNΔ7* mRNA levels in GM03813 SMA fibroblasts treated with different doses of AR42, AR19, 4PBA and TSA for 5 days as well as in GM03814 healthy carrier fibroblasts. The *SMNΔ7* mRNA levels were normalized to those of DMSO-treated GM03813 SMA fibroblasts (dashed line). (**E**) Changes in SMN protein levels in GM03813 SMA fibroblasts treated with different doses of AR42, AR19, 4PBA and TSA for 5 days as well as in GM03814 healthy carrier fibroblasts. The SMN protein levels are relative to those observed for β-actin. The data are presented as mean ± standard error. The asterisk (*) denotes a statistically significant (*p* ≤ 0.05; one-way ANOVA with Bonferroni post hoc test) difference between drug- and vehicle-treated GM03813 SMA cells.

To further investigate how *SMN2* expression is regulated by AR42, we examined the effect of AR42 on *SMN2* promoter activity using NSC34 motor neuron-like cells stably transfected with a β-lactamase reporter gene driven by a 3.4-kb fragment of the *SMN2* promoter (clone 11)^41^. In response to exposure to 100 nM AR42, *SMN2* promoter activity was increased by 54%; TSA also induced *SMN2* promoter activity by 74% at this dose (Fig. 3A). Neither 4PBA nor AR19 affected *SMN2* promoter activity at this dose. AR42 (Fig. 3B) increased *SMN2* promoter activity in NSC34 cells in a dose-dependent manner with an estimated EC_50_ of 24 nM (95% confidence interval (CI) = 11.98–83.62 nM; R^*2*^ = 0.9372).

The effects of these compounds on the expression of *SMN2* were examined in GM03813 type II SMA fibroblasts. Cells were treated with different doses of AR42, AR19, 4PBA and TSA for 5 days. Surprisingly, AR42 had no effect on either *FL-SMN* (Fig. 3C) or *SMNΔ7* (Fig. 3D) mRNA levels even though these transcripts were induced in SMA fibroblasts treated with 4PBA or TSA. Treatment of GM03813 cells with AR19 did not increase expression of *FL-SMN* or *SMNΔ7* mRNAs. Analysis of SMN protein levels in treated GM03813 type II SMA fibroblasts showed the same patterns as those for *FL-SMN* mRNA changes in response to drug treatment (Fig. 3E). Thus, AR42 does not upregulate *SMN2* mRNA or protein expression but increases the subnuclear localization of SMN in SMA fibroblasts.

### Administration of AR42 before onset of motor neuron loss improved the survival of and delayed disease end-stage in SMNΔ7 SMA mice

**Figure 4:**
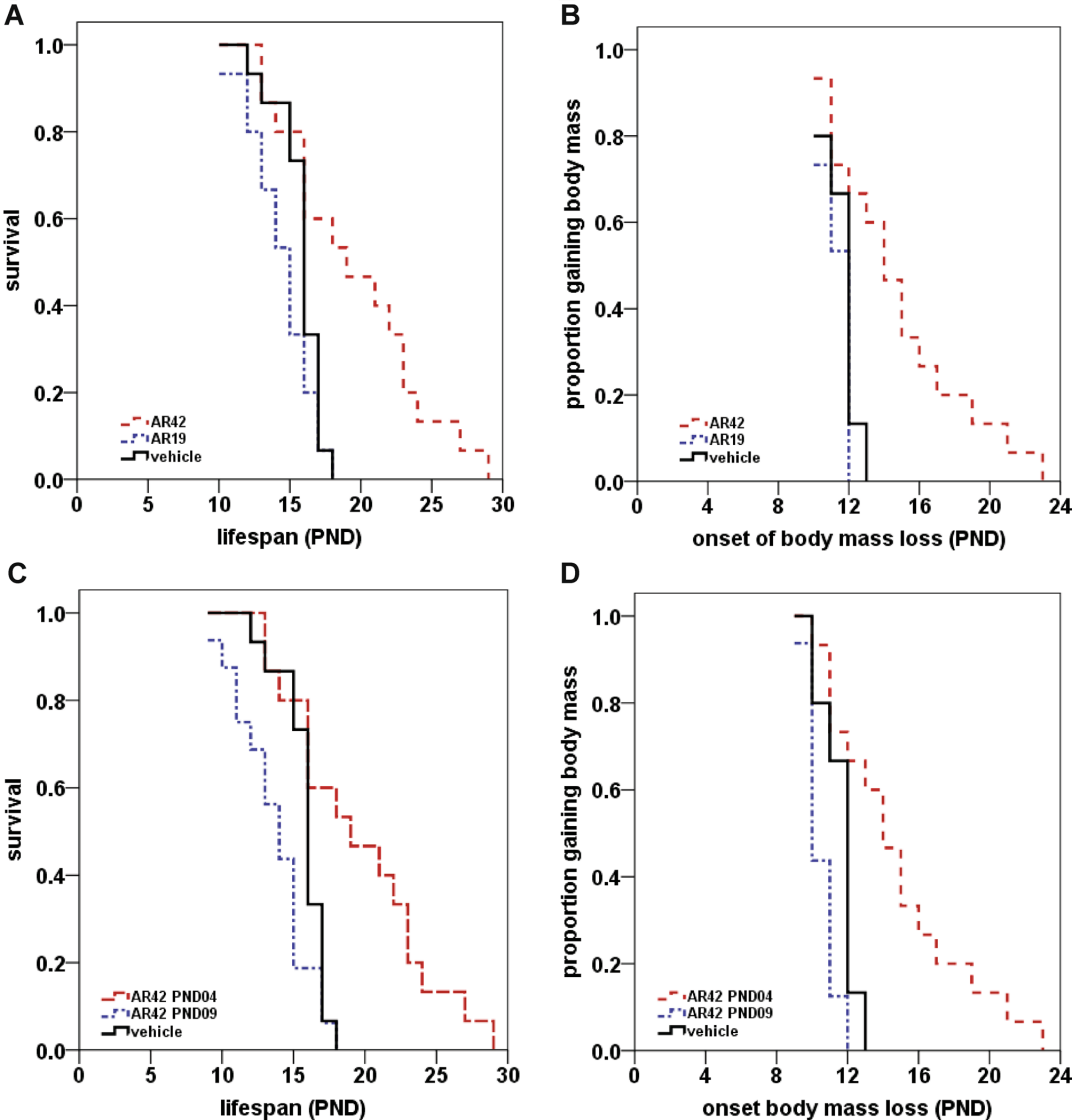
Effects of AR42 and AR19 on the lifespan and disease end-stage in SMNΔ7 SMA mice. (**A**, **B**) SMNΔ7 SMA mice were treated daily with AR42 (5 mg/kg/d; red dashed line) or AR19 (5 mg/kg/d; blue dotted line) beginning at PND04 and monitored for changes in lifespan (**A**) and disease end-stage, which is defined as the onset of body mass loss (**B**). AR42 increased survival by 27% (**A**; *p* = 0.011; Kaplan–Meier analysis with Mantel–Cox log rank post hoc test) and delayed the onset of body mass loss by 31% (**B**; *p* = 0.002; Kaplan–Meier analysis with Mantel–Cox log rank post hoc test). AR19, on the other hand did not affect lifespan or disease end-stage in SMNΔ7 SMA mice. (**C**, **D**) SMNΔ7 SMA mice were treated with AR42 (5 mg/kg/d) daily beginning at either PND04 (red dashed line) or PND09 (blue dotted line). Post-symptomatic—i.e. beginning at PND09, or after onset of motor neuron loss—treatment with AR42 did not positively affect survival (**C**) or onset of body mass loss (**D**) in these mice. In fact, post-symptomatic AR42 treatment resulted in earlier death of SMNΔ7 mice than vehicle (black line).

AR42 is orally bioavailable and can cross the blood–brain barrier^42^. To determine if AR42 treatment has a protective effect on the SMA phenotype, SMNΔ7 SMA mice were treated with AR42 (5 mg/kg/day; n = 15) or vehicle (n = 15) beginning at PND04. As a control, SMNΔ7 SMA mice were treated with the less potent analogue AR19 (5 mg/kg/day; n = 15). In a pilot dose determination trial, we found that neonatal mice treated with higher doses of AR42 (≥ 10 mg/kg/day) showed off-target toxicity like significant loss in body mass after dosing and reduced locomotion (data not shown). Bjornsson et al.^43^ also observed off-target effects in a mouse model for Kabuki syndrome that was treated with higher doses of AR42. As shown in Fig. 4A, oral administration of AR42 improved the survival of SMNΔ7 SMA mice by 27% (20.1 ± 1.6 days for AR42-treated mice as compared to 15.8 ± 0.4 days for vehicle-treated mice; *p* = 0.011, χ^2^ = 6.453). Treatment of SMNΔ7 SMA mice with AR19, however, did not significantly affect survival (14.5 ± 0.6 days for AR19-treated mice as compared to 15.8 ± 0.4 days for vehicle-treated mice; *p* = 0.132, χ^2^ = 2.264).

In the SMNΔ7 SMA mouse model, a phenotypic indicator of disease end-stage is the onset of loss of body mass^13,44^. The onset of body mass loss was delayed by 31% (Fig. 4B; 14.8 ± 1.0 days for AR42-treated mice as compared to 11.6 ± 0.3 days for vehicle-treated mice; *p* = 0.002, χ^2^ = 9.616) in SMNΔ7 SMA mice treated with AR42. Treatment of SMNΔ7 SMA mice with AR19, however, did not affect the onset of body mass loss in these mice (Fig. 4B; 11.3 ± 0.2 days for AR19-treated mice as compared to 11.6 ± 0.3 days for vehicle-treated mice; *p* = 0.239, χ^2^ = 1.389).

Therapeutic efficacy in SMA mouse models depends on the timing of treatment. For example, the DcpS inhibitor D156844^45^ and the butyrate prodrug VX563^36^ ameliorate the degenerative phenotype in SMNΔ7 SMA mice only if treatment begins before the onset of motor neuron loss. We, therefore, treated SMNΔ7 SMA mice with AR42 (n = 16) starting at postnatal day 9 (PND09) and did not observe an improvement in their survival (Fig. 4C); in fact, these mice died on average before vehicle-treated SMNΔ7 SMA mice (13.7 ± 0.7 days for AR42-treated mice at PND09 as compared to 15.8 ± 0.4 days for vehicle-treated mice; *p* = 0.043, χ^2^ = 4.103). Post-symptomatic treatment of SMNΔ7 SMA mice also negatively impacted disease end-stage (Fig. 4D; 10.5 ± 0.2 days for AR42-treated mice at PND09 as compared to 11.6 ± 0.3 days for vehicle-treated mice; *p* = 0.002, χ^2^ = 9.714). Taken together, these observations show therapeutic efficacy of AR42 in SMNΔ7 SMA mice when treatment with this drug begins prior to disease onset.

### Effect of AR42 on the phenotype of SMNΔ7 SMA mice

**Figure 5:**
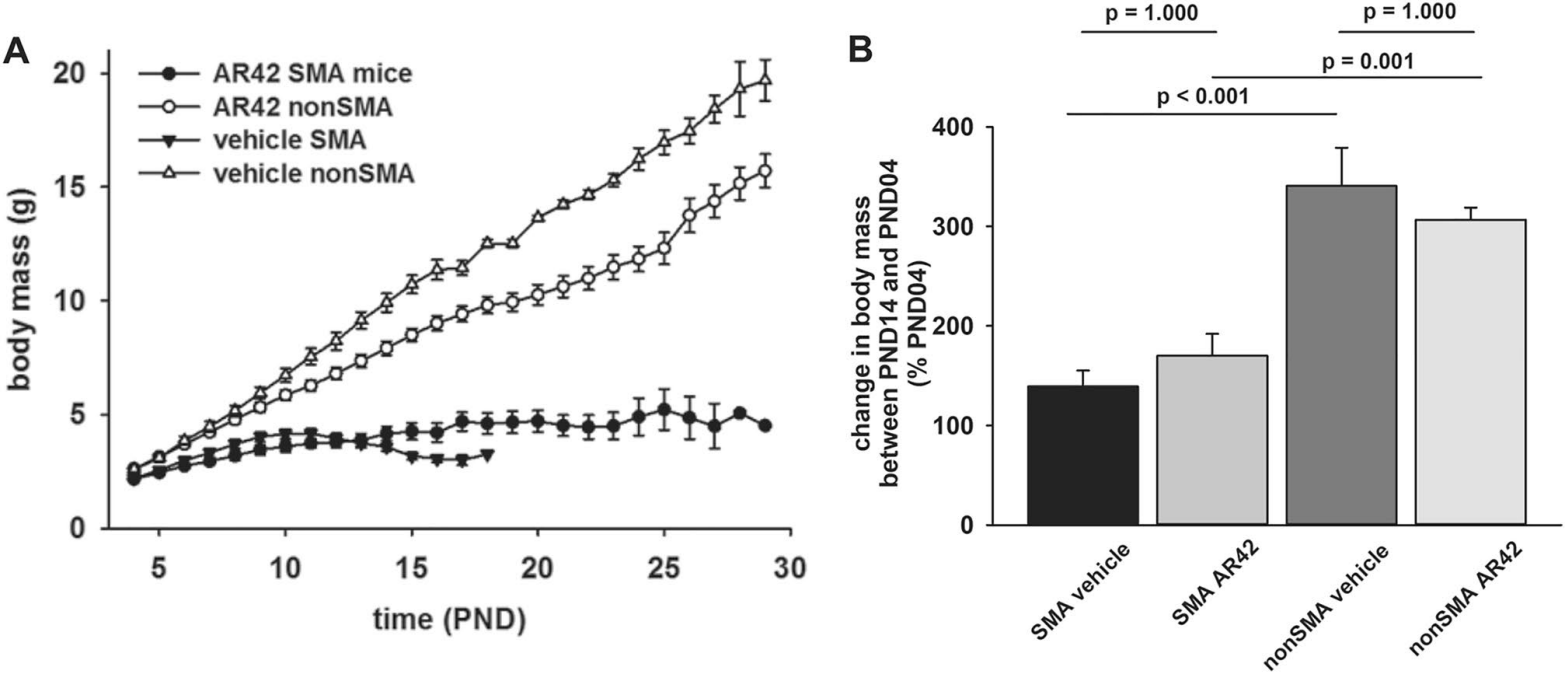
Effect of AR42 on the body mass of SMNΔ7 SMA mice over time. (**A**) Body mass curves of SMNΔ7 SMA mice (solid shapes) or non-SMA (either carrier or normal; open shapes) littermates treated daily with either AR42 (5 mg/kg/d; circles) or vehicle (triangles). (**B**) Changes in body mass between PND14 and PND04 in SMNΔ7 SMA mice treated with AR42 or vehicle along with non-SMA littermates. The mean body masses at PND14 were expressed relative to those at PND04. The data are presented as mean ± standard error. The asterisk (*) denotes a statistically significant (*p* ≤ 0.05; one-way ANOVA with Bonferroni post hoc test) difference when comparing SMNΔ7 SMA and non-SMA mice for each treatment group.

As an indicator of growth rate in developing mice, we monitored the changes in body mass over time in SMNΔ7 SMA and non-SMA littermates treated with AR42 or vehicle. Consistent with previous findings using this colony of mice^36,44–48^, SMNΔ7 SMA mice had lower body masses than their age-matched non-SMA littermates regardless of treatment (Fig. 5A). However, the body masses of AR42-treated SMNΔ7 SMA were greater than age-matched vehicle SMNΔ7 SMA mice (Fig. 5A) especially at the late-stage of disease (i.e. PND15–17) but these differences were not statistically significant. When comparing the body masses between PND14 and PND04, AR42-treated SMNΔ7 SMA mice tended to show a small increase in the change in body mass when compared to vehicle-treated SMNΔ7 SMA mice but the difference was not statistically significant (Fig. 5B). We also observed a slight reduction in body mass of nonSMA mice treated with AR42 but the change was not statistically significant (*p* = 0.325).

**Figure 6:**
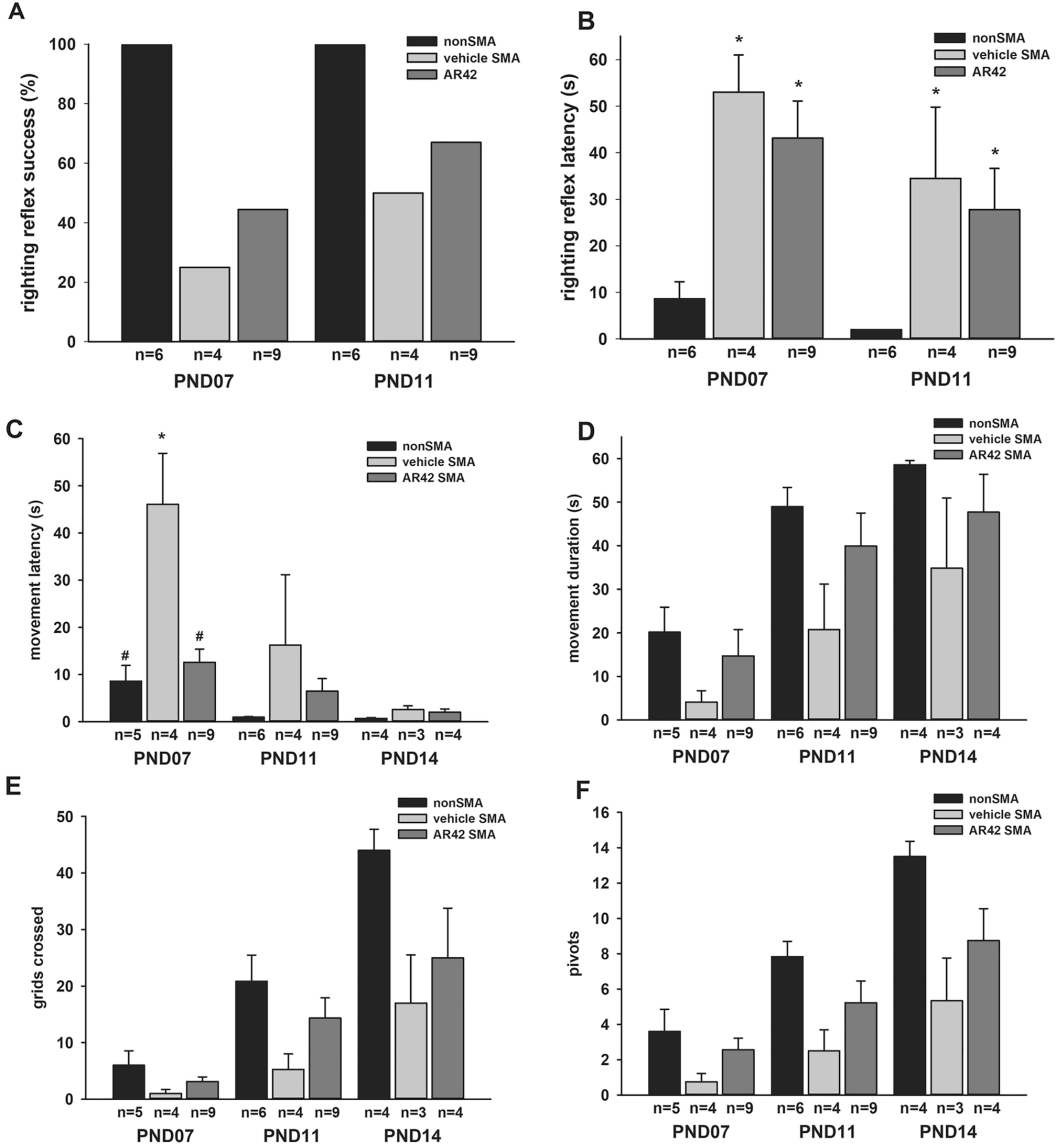
Effect of AR42 on the motor phenotype of SMNΔ7 SMA mice. SMNΔ7 SMA mice were treated either with AR42 (5 mg/kg/d) or vehicle beginning at PND04 and monitored at PND07, PND11 and PND14 for changes in motor behavior. Age-matched, non-SMA littermates were also assayed at each time point. (**A**) SMNΔ7 SMA mice showed impaired surface righting responses at PND07 and PND11 relative to age-matched control littermates. A greater proportion of AR42-treated SMNΔ7 SMA mice exhibited a successful surface righting response than vehicle-treated SMNΔ7 SMA mice. (**B**) SMNΔ7 SMA mice treated with either AR42 or vehicle had a higher righting reflex latency than control mice at both ages. The righting reflex latency time has an arbitrary cutoff of 61 s. (**C**) The latency of vectorial movement, or locomotion along a single vector for a distance greater than the body length, was higher in vehicle-treated SMNΔ7 SMA mice than control mice at PND07 and PND11. AR42 treatment markedly reduced vectorial movement latency in SMNΔ7 SMA mice when compared against vehicle-treated SMNΔ7 SMA mice. (**D**) Vectorial movement duration was reduced in vehicle-treated SMNΔ7 SMA mice when compared against age-matched control mice. AR42 tended to increase vectorial movement duration in SMNΔ7 SMA mice at PND07 and PND11. (**E**) SMNΔ7 SMA mice crossed fewer grids within 60 s, a measure of spontaneous locomotor activity, than control mice. AR42 treatment of SMNΔ7 SMA mice increased spontaneous locomotor activity relative to vehicle-treated SMNΔ7 SMA mice at PND11 and PND14 but the differences were not statistically significant. (**F**) The number of 90° pivots made in 60 s was reduced in vehicle-treated SMNΔ7 SMA mice when compared against age-matched control littermates. AR42 treatment of SMNΔ7 SMA mice increased the pivoting response relative to vehicle-treated SMNΔ7 SMA mice at all ages but the differences were not statistically significant. The data are presented as mean ± standard error. The asterisk (*) denotes a statistically significant (*p* ≤ 0.05; one-way ANOVA with Bonferonni post hoc test) difference when compared to control mice and the number sign (#) denotes a statistically significant (*p* ≤ 0.05; one-way ANOVA with Bonferonni post hoc test) difference when compared to vehicle-treated SMNΔ7 SMA mice.

AR42 treatment before the onset of motor neuron loss improved the motor phenotype observed in SMNΔ7 SMA mice (Supplementary Movie). The effect of AR42 on motor impairment observed in the SMNΔ7 SMA mice was examined in further detail. SMNΔ7 SMA mice were treated with AR42 (5 mg/kg/d) or vehicle beginning at PND04. The controls for this experiment were unaffected (both carrier and normal pups) littermates; the motor phenotypes between carrier and normal mice were nearly identical and, hence, were combined in this experiment^44^. Since sex does not affect the motor phenotype in SMNΔ7 SMA mice^44^, both male and female pups were used. The success rate for the righting reflex was higher in SMNΔ7 SMA mice treated with AR42 than vehicle-treated SMNΔ7 SMA mice at PND07 and PND11 (Fig. 6A) but the righting reflex latencies were not altered by AR42 treatment (Fig. 6B). Vectorial movement is defined as locomotion along a single vector for a distance greater than the body length^44^. AR42 treatment markedly reduced vectorial movement latency in SMNΔ7 SMA mice relative to vehicle (Fig. 6C); in fact, the vectorial movement latency for AR42-treated SMNΔ7 SMA mice was similar to control littermates. The duration of vectorial movement tended to be higher in AR42-treated SMNΔ7 SMA mice than in vehicle-treated SMNΔ7 SMA mice at PND07 and PND11 but the difference was not statistically significant (Fig. 6D). AR42 treatment of SMNΔ7 SMA mice tended to increase spontaneous locomotor activity—the number of grids crossed in 60 s—relative to vehicle-treated SMNΔ7 SMA mice at PND11 and PND14 (Fig. 6E). AR42 treatment of SMNΔ7 SMA mice increased the pivoting response, i.e. the number of 90° pivots made in 60 s, relative to vehicle-treated SMNΔ7 SMA mice at all ages but the differences were not statistically significant (Fig. 6F). As we have observed in previous preclinical drug trials with the SMNΔ7 SMA mice^36,48^, there was a high degree of variability within the motor phenotype of each treatment group—especially at PND14—which could indicate strong and weak responders to AR42.

### Effect of AR42 on motor neuron loss in the mouse lumbar spinal cord of SMNΔ7 SMA mice

**Figure 7:**
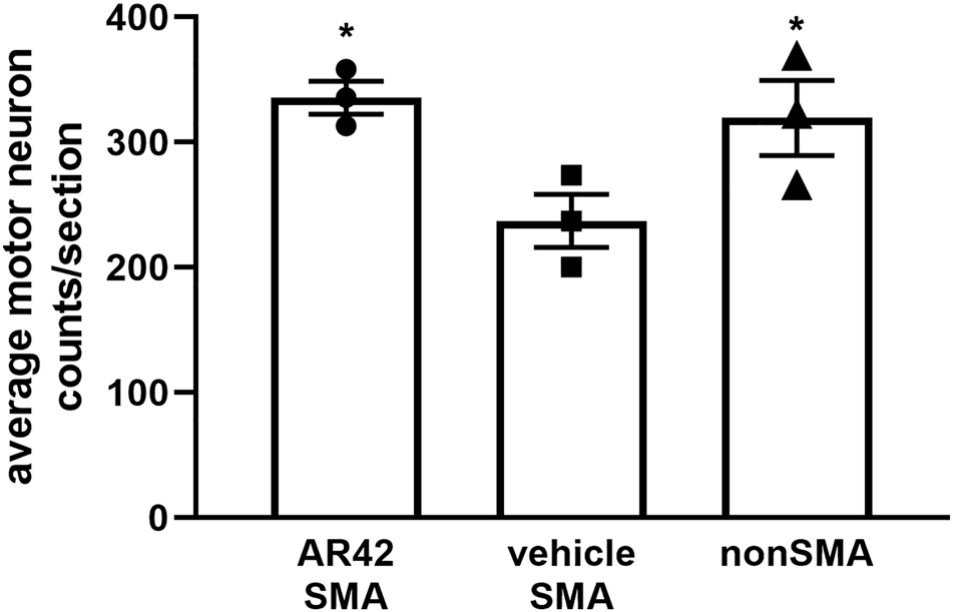
Effect of AR42 on motor neuron loss in the lumbar spinal cords of SMNΔ7 SMA mice. SMNΔ7 SMA mice (n = 3/group) were treated with either AR42 or vehicle from PND04 until PND11. The number of Cresyl violet-positive, ventral motor neurons were counted in every 12th section of lumbar spinal cords of AR42-treated SMNΔ7 SMA mice (circles), vehicle-treated SMNΔ7 SMA mice (squares) or age-matched non-SMA littermates (triangles). AR42 increased lumbar spinal motor neuron counts in SMNΔ7 SMA mice to levels similar to those in nonSMA littermates. The data are presented as mean ± standard error with the average motor neuron counts/section shown for each individual mouse. The asterisk (*) denotes a statistically significant (*p* ≤ 0.05; one-way ANOVA with Bonferonni post hoc test) difference when compared to vehicle-treated SMNΔ7 SMA mice.

Significant motor neuron loss in the lumbar spinal cord was observed in the SMNΔ7 SMA mice at PND11^13^. Treatment of SMNΔ7 SMA mice with 4-PBA significantly rescued motor neuron loss at PND11^36^. Given the improvement of motor function in SMNΔ7 SMA mice treated with AR42, we examined the effect of AR42 on motor neuron loss in the spinal cords of SMNΔ7 SMA mice. We observed a 29.8% reduction in the number of motor neurons in the lumbar spinal cord of SMNΔ7 SMA mice at PND11 (Fig. 7), similar to the reduction in motor neuron counts we observed in previous studies^36,45^. AR42 treatment leads to an increase in motor neuron counts in SMNΔ7 SMA mouse lumbar spinal cords to counts similar to those observed in non-SMA mice at PND11.

### Effect of AR42 on SMN2 expression in the spinal cord of SMNΔ7 SMA mice

**Figure 8:**
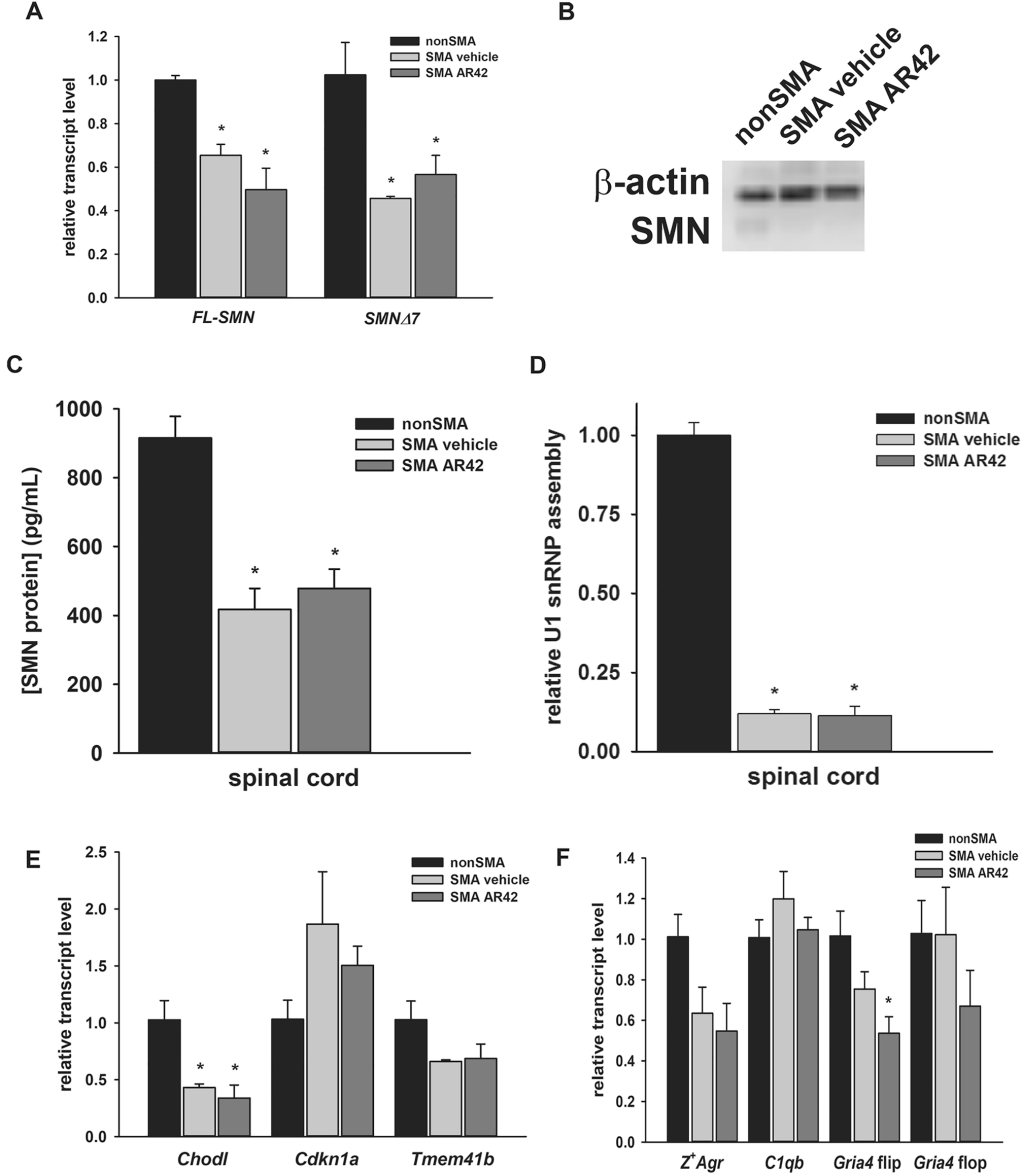
Effect of AR42 on SMN protein levels and function in the spinal cords of SMNΔ7 SMA mice. SMNΔ7 SMA mice (n = 3/group) were treated with either AR42 or vehicle for 5 days beginning at PND04. (**A**) AR42 did not affect *FL-SMN* or *SMNΔ7* mRNA levels in the spinal cords of SMNΔ7 SMA mice. (**B**) Total SMN protein levels in the spinal cord were not altered by AR42 treatment as shown by this representative immunoblot. (**C**) Quantification of human SMN protein using ELISA also showed no significant changes between AR42- and vehicle-treated SMNΔ7 SMA mice but SMN protein levels were lower in SMNΔ7 SMA mice when compared to age-matched non-SMA littermates. (**D**) In vitro assembly of U1 snRNPs was diminished in SMNΔ7 SMA spinal cord samples but AR42 treatment did not affect snRNP assembly. (**E**) The levels of the SMN-dependent transcripts *Chodl*, *Cdkn1a* and *Tmem41b* were not affected by AR42 treatment in SMNΔ7 SMA spinal cords. (**F**) AR42 significantly reduced the mRNA levels of the flip, but not the flop, isoform of *Gria4* in SMNΔ7 SMA spinal cords. The levels of the synaptogenesis-associated transcripts *Z*^+^*Agr* and *C1qb* were not affected by AR42. The data are presented as mean ± standard error. The asterisk (*) denotes a statistically significant (*p* ≤ 0.05; one-way ANOVA with Bonferonni post hoc test) difference when compared to vehicle-treated carrier mice.

We wanted to determine the effect of AR42 on SMN expression in vivo in the spinal cords of SMNΔ7 SMA mice. These mice were treated with AR42 (5 mg/kg/d) or vehicle (n = 3/group) beginning at PND04, and treatment continued for 5 days (until PND08). Treatment of SMNΔ7 SMA mice with AR42 did not increase the levels of *FL-SMN* or *SMNΔ7* mRNAs in the spinal cord (Fig. 8A). As shown in Fig. 8B, AR42 treatment did not significantly affect SMN protein levels in the spinal cord of treated SMNΔ7 SMA mice. SMN ELISA analysis also showed no change in SMN protein levels in response to AR42 treatment in SMNΔ7 SMA mice (Fig. 8C). Spliceosomal and U7 snRNP assembly are well established functions of SMN at the molecular level^49^ that are impaired in SMA mouse models^50–52^ and AR42 treatment did not affect snRNP assembly in spinal cord extracts from SMNΔ7 SMA mice (Fig. 8D).

We examined the effect of AR42 on the levels of 3 different SMN-dependent transcripts: *chondrolectin* (*Chodl*), *cyclin-dependent kinase inhibitor 1A* (*Cdkn1a*) and *transmembrane protein 41b* (*Tmem41b*), the murine orthologue to the *Drosophila* gene *stasimon*^53–55^. *Chodl* levels were significantly lower in SMNΔ7 SMA spinal cords at PND08 and there were tendencies for increased *Cdkn1a* and reduced *Tmem41b* mRNA levels in these samples as well (Fig. 8E). AR42, however, did not affect the expression of these SMN-dependent transcripts in the SMNΔ7 SMA spinal cord.

In motor neurons, SMN deficiency leads to an early dysregulation of transcripts that are involved in synaptogenesis^56^. The effects of AR42 on the dysregulation of 4 of these synaptogenesis-associated transcripts—*Z*^+^ *agrin* (*Z*^+^*Agr*), *complement component 1 B polypeptide* (*C1qb*) as well as the *AMPA-type ionotropic glutamate receptor 4* (*Gria4*) alternatively spliced flip and flop isoforms—in SMNΔ7 SMA spinal cords. We observed trends towards reduction of *Z*^+^*Agr* mRNA levels and increase of *C1qb* mRNA levels in SMNΔ7 SMA spinal cords at PND08; however, AR42 had no effect on their expression (Fig. 8F). At PND08, we observed a decrease in *Gria4* flip mRNA levels but no change in the levels of the flop isoform. AR42 treatment further reduced *Gria4* flip mRNA levels in the SMNΔ7 SMA spinal cord (Fig. 8F). Based on these observations, we conclude that AR42—like 4PBA and the butyrate-based prodrug VX563^36^—mediates its neuroprotective effects in the spinal cord independent of modifying *SMN2* expression and SMN-dependent gene regulation.

### Effects of AR42 on HDAC activity in SMNΔ7 SMA mice

**Figure 9:**
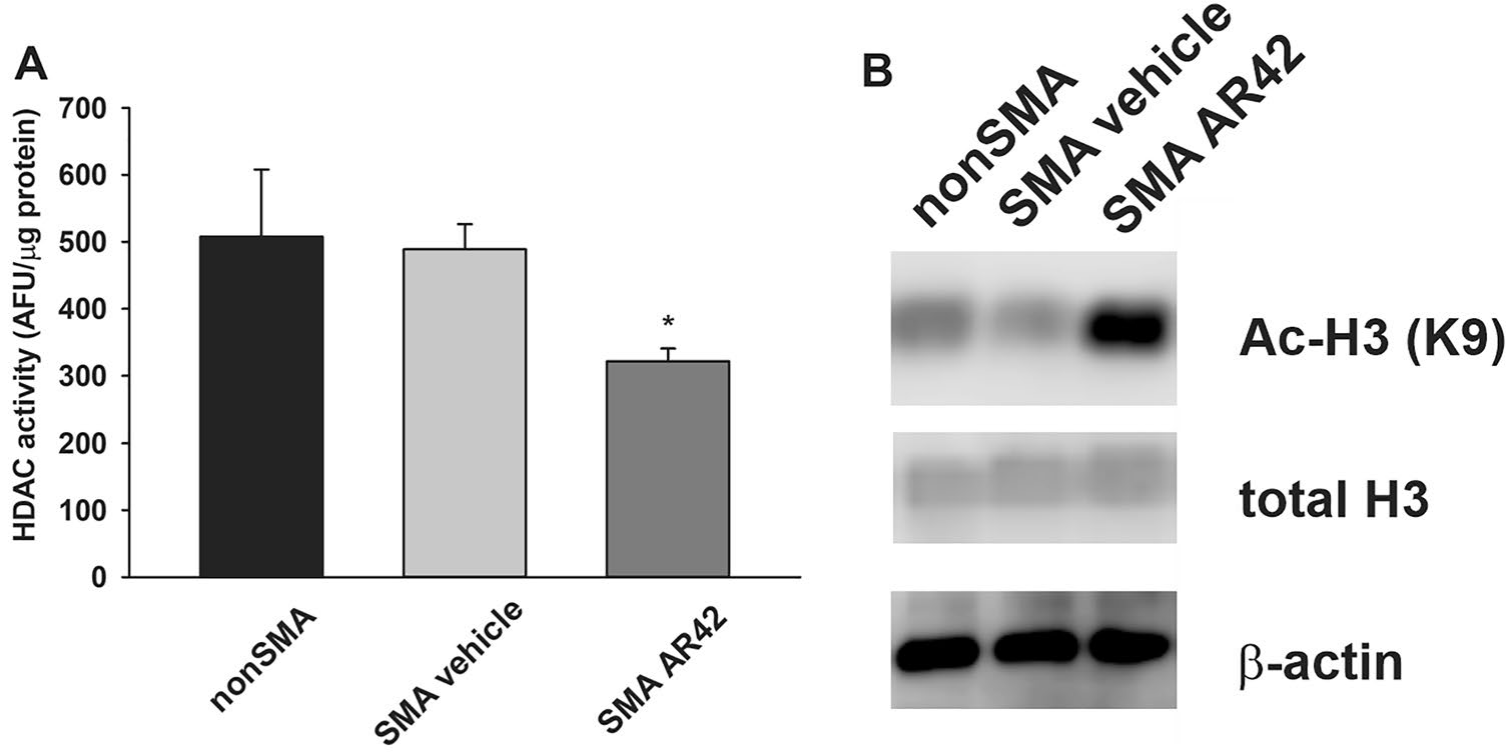
Effect of AR42 on HDAC activity in the spinal cords of SMNΔ7 SMA mice. SMNΔ7 SMA mice (n = 3/group) were treated with either AR42 or vehicle for 5 days beginning at PND04. As a control, non-SMA mice (n = 3) were treated with vehicle for 5 days beginning at PND04. (**A**) Fluorimetric HDAC activity in treated spinal cord. Treatment of SMNΔ7 SMA mice with AR42 significantly reduced HDAC enzyme activity in the spinal cord. The data are presented as mean ± standard error. The asterisk (*) denotes a statistically significant (*p* ≤ 0.05; one-way ANOVA with Bonferonni post hoc test) difference when compared to vehicle-treated SMNΔ7 SMA mice. (**B**) Representative immunoblots of acetyl-histone H3 (K9) and total histone H3 levels in treated spinal cords. All band intensities were expressed relative to β-actin band intensities.

As AR42 has very potent HDAC inhibitory activity^37,38^, spinal cord extracts from SMNΔ7 SMA mice treated with AR42 (5 mg/kg/d) or vehicle for 5 days were assayed for HDAC activity. Fluorimetric HDAC activity was lower in spinal cord samples from SMNΔ7 SMA mice treated with AR42 than those mice treated with vehicle (Fig. 9A). We also observed a marked increase in histone H3 acetylation at K9 in SMNΔ7 SMA spinal cords from AR42-treated mice (Fig. 9B). Interestingly, HDAC activity and histone acetylation were not different in spinal cords from vehicle-treated SMNΔ7 SMA mice and healthy controls.

### Effect of AR42 on Akt signaling in SMNΔ7 SMA mice

**Figure 10:**
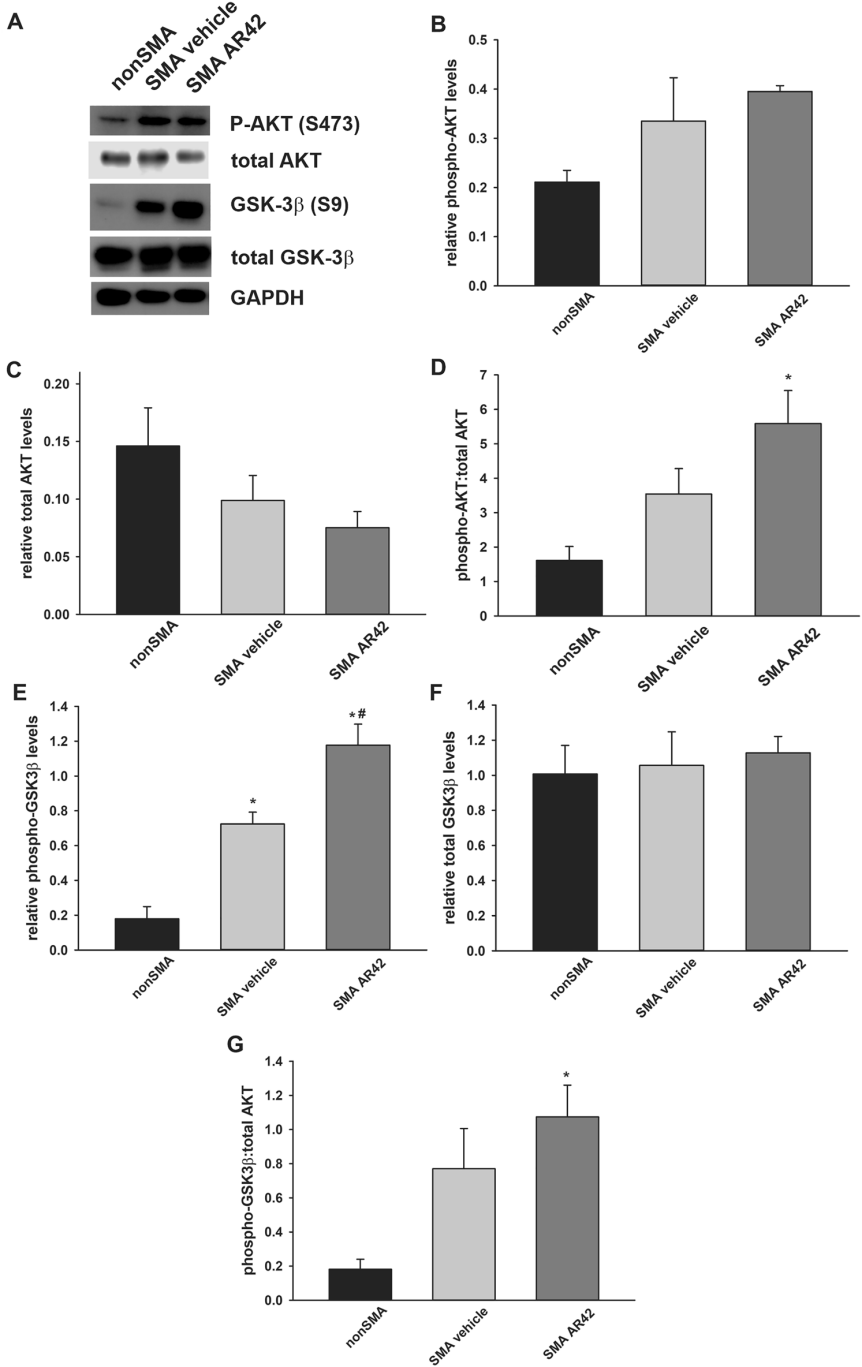
Effect of AR42 on AKT and GSK3β phosphorylation in the spinal cords of SMNΔ7 SMA mice. SMNΔ7 SMA mice (n = 3/group) were treated with either AR42 or vehicle for 5 days beginning at PND04. As a control, non-SMA mice (n = 3) were treated with vehicle for 5 days beginning at PND04. (**A**) Representative immunoblots of phosphorylated Akt (S473), total Akt, phosphorylated GSK3β (S9) and total GSK3β levels in treated spinal cords. All band intensities were expressed relative to GAPDH band intensities. (**B**) There is a trend for increased phosphorylation of Akt at S473 in SMNΔ7 SMA mouse spinal cords that was not affected by AR42 treatment. (**C**) Total Akt protein levels were significantly increase in SMA spinal cords but AR42 treatment reduced total Akt protein levels to control. (**D**) The Akt (phospho-S473) versus total Akt ratio was significantly higher in the spinal cords of SMNΔ7 SMA mice treated with AR42. (**E**) Phosphorylation of GSK3β at S9 was significantly increased in SMNΔ7 SMA mouse spinal cords and treatment with AR42 further elevated GSK3β at S9. (**F**) The levels of total GSK3β protein were not altered by SMA genotype nor by AR42 treatment. (**G**) The GSK3β (phospho-S9) versus total GSK3β ratio was significantly higher in the spinal cords of SMNΔ7 SMA mice treated with AR42. The data are presented as mean ± standard error. The asterisk (*) denotes a statistically significant (*p* ≤ 0.05; one-way ANOVA with Bonferonni post hoc test) difference when compared to vehicle-treated carrier mice and the number symbol (#) denotes a statistically significant (*p* ≤ 0.05; one-way ANOVA with Bonferonni post hoc test) difference when compared to vehicle-treated SMA spinal cords.

Previous work in cancer cells^57^ identified a link between AR42-mediated HDAC inhibition and Akt signaling. Based on these findings, we measured Akt and GSK3β—a downstream target of Akt—phosphorylation status in SMNΔ7 SMA mice treated with AR42 for 5 days. Phosphorylation of Akt at S473 in SMNΔ7 SMA mice was elevated relative to unaffected control mice, but the differences were not statistically significant (*p* = 0.064; Fig. 10A, B). Treatment with AR42 did not significantly increase Akt phosphorylation at serine 473 (S473) in SMA spinal cord relative to vehicle treated SMNΔ7 mice. Interestingly, the levels of total Akt protein were diminished in the spinal cords of SMNΔ7 SMA mice relative to control mice and AR42 treatment reduced total AKT protein levels to those observed in control mice although the changes were not statistically significant (Fig. 10C). The Akt (phospho-S473) versus total Akt ratio was significantly higher in the spinal cords of SMNΔ7 SMA mice treated with AR42 than in vehicle-treated SMNΔ7 SMA mice as well as compared against control mice (Fig. 10D).

SMNΔ7 SMA mouse spinal cords showed elevated phosphorylation of GSK3β at serine 9 (S9) when compared against control mice (Fig. 10A, E). Treatment of SMNΔ7 SMA mice with AR42 further increased GSK3β phosphorylation at S9 in the spinal cord (Fig. 10E). Total GSK3β levels were not affected by SMA status or by AR42 treatment (Fig. 10F). The GSK3β (phospho-S9) versus total GSK3β ratio was significantly higher in the spinal cords of SMNΔ7 SMA mice treated with AR42 than in control mice (Fig. 10G). Taken together, our data demonstrate that AR42 increases Akt and GSK3β phosphorylation in the spinal cords of SMNΔ7 SMA mice.

## Discussion

In this study, we demonstrate in vivo efficacy of a novel, orally bioavailable HDAC inhibitor, AR42, in a mouse model for SMA^37,38,42^. AR42 is a 4PBA-tethered TSA derivative^37,38^. AR42 functions as pan-inhibitor of class I HDACs, i.e. HDAC1, HDAC2, HDAC3 and HDAC8^58^. Similar to our findings that 4PBA and BA prodrugs ameliorate the survival and phenotype of SMNΔ7 SMA^36,59^, other HDAC inhibitors including VPA^60^, TSA^26,34,35^ and SAHA^28^ have been found to improve survival in mouse models for SMA. Treatment of SMA mice with JNJ-26481585 does not significantly improve their survival but there is a trend for amelioration of their motor phenotype^31^. While many of these studies showed increased SMN expression in vivo in response to HDAC inhibitor treatment^26,28,35,60^, we did not observe any detectable changes in SMN mRNA or protein levels in SMNΔ7 SMA mice treated with AR42. Consistent with our findings, Liu et al.^34^ also demonstrated that the beneficial effects of TSA treatment in SMA mice are independent of SMN upregulation. Moreover, in a clinical trial, SMA patients treated with VPA do not show increased SMN expression even though VPA treatment results in increased histone acetylation^61^. In addition to their neuroprotective roles, HDAC inhibitors like SAHA and TSA exert extra-neuronal protective actions in SMA mice like increasing muscle microvasculature^62^ and reducing levels of atrogene transcripts in affected muscle^63^.

As observed with AR42, some small molecules which increase *SMN2* promoter activity do not necessarily increase *FL-SMN* transcript levels or SMN protein levels. This disconnect may be a consequence of the *SMN2* promoter screening assay itself as the construct used may not fully represent the endogenous promoter activity. Alternatively, AR42 may be acting at the post-transcriptional/translational levels of SMN regulation. Compounds including ML372, LDN-75654 and cuspin-1 upregulate SMN expression post-transcriptionally, when validated in SMA patient-derived cell lines^64–66^. Flunarizine as well as the C5-substituted, 2,4-diaminoquinazolines D156884 and D157495 increase SMN localization to subnuclear gems without markedly increasing SMN expression^67,68^. D156844 and D157495 significantly improve the survival and motor phenotypes of SMA mice^45,69,70^. While SMN expression was not significantly altered in the spinal cord of SMA mice treated with these compounds, the subcellular localization of SMN in SMA motor neurons in vivo may have been affected. By extension, AR42 may increase the subcellular localization of SMN in neurons in vivo without increasing SMN expression. It is equally possible that the protective mode of action for AR42 in SMA mice may be independent of *SMN2* gene regulation.

Since upregulation of SMN expression is not the primary mode of action for AR42 in SMA mice, we determined that the protective effects of AR42 may be related to differential phosphorylation of Akt and GSK3β. The Akt pathway is neuroprotective in neurons when its activity is increased^71^. Previous studies have shown that Akt phosphorylation is reduced in the spinal cords of symptomatic SMA mice^72,73^. In this study, we show that phosphorylation of Akt at S473 is increased in SMNΔ7 SMA mouse spinal cords and AR42 further potentiates that elevation in Akt phosphorylation at S473.

One of the primary targets of Akt activation is GSK3β. Inhibition of GSK3α/β activity with alsterpaullone increase SMN protein levels in vitro by reducing the rate of degradation in SMA fibroblasts and in *Smn*-deficient mESC-derived motor neurons^74^. BIP-135, a GSK3α/β inhibitor, increases survival of SMNΔ7 SMA mice by 15%^75^. In this study, we demonstrate that AR42 treatment increases GSK3β phosphorylation at S9 in the SMNΔ7 SMA mouse spinal cord. Phosphorylation of GSK3β at S9 by Akt inhibits the activity of GSK3β^76^. GSK3β inactivation by increasing its phosphorylation at S9 is essential for the development of and maintenance of neuronal polarity in neurons^77–79^. AR42 may protect the surviving SMA motor neurons from degeneration by enhancing inactivation of GSK3β through phosphorylation by Akt.

AR42 has shown strong potential as a therapeutic option in preclinical animal studies as well as in clinical trials for different forms of cancer. AR42 shows strong efficacy as a tumor growth inhibitor in multiple in vivo models for cancer progression^42,80–84^. Tumor-induced loss of muscle mass and function, i.e. cachexia, is diminished by AR42^85^. AR42 has recently demonstrated clinical efficacy in phase I trials for acute myeloid leukemia, multiple myeloma, T-cell lymphomas, NF2 schwannomas and meningiomas^86–89^. In addition to its role as a potential cancer therapeutic, AR42 also improves hippocampal memory deficits present in a transgenic mouse model for Kabuki syndrome^43^.

While AR42 treatment does increase survival and delay spinal motor neuron loss in SMNΔ7 SMA mice, these mice still display a motor phenotype. Previous studies have shown that SMA mice have additional deficits in the spinal sensory-motor circuit^90–93^. It is possible that AR42 improves motor neuron survival in SMA, which extends survival and improves motor behavior, but does not significantly ameliorate these sensory-motor circuit deficits. Previous work has shown that coadministration of small molecule circuit modifier—4-aminopyridine—and a neuroprotectant agent—pifithrin-α—provided significant therapeutic benefit to SMA mice^94^. Combination therapeutic regimens have previously shown additive therapeutic benefit in SMA animal models^48,95–97^. Future studies will explore the utility of AR42 as part of a multi-modal and multi-indication therapy for SMA as well as for other motor neuron diseases.

In conclusion, we have demonstrated that the class I HDAC inhibitor AR42 significantly improves the survival and motor phenotype in SMNΔ7 SMA mice in a SMN-independent manner when administered presymptomatically. AR42 increases AKT signaling in SMA spinal cords which may provide a neuroprotective mechanism of action for this compound. Future studies will characterize the regulation of AKT signaling in SMA motor neurons to determine a novel protective therapeutic strategy for SMA. While the currently approved therapies^14–17^ have significantly improved the prognosis for SMA patients, they do not fully ameliorate the disease phenotype in SMA patients. *SMN2*-independent therapies could further augment motor function in SMA patients. There are two biologics—apitegromab (SRK-015) and reldesemtiv (CK-2127107)—targeting the improvement of muscle function that are currently undergoing clinical trials in SMA patients^98,99^. Small molecule HDAC inhibitors like 4PBA^36^, VX-563^36^ and AR42 (this study) offer another SMN-independent protective strategy that could be used in concert with *SMN2* inducers to maximize therapeutic outcomes in SMA patients. The additive effects of HDAC inhibition on the efficacy of *SMN2* inducers has been recently demonstrated by the enhanced neuroprotective effects of valproic acid and splice-correcting antisense oligonucleotides^96^.

## Methods

### Drug compounds

AR42 (OSU-42; HDAC-42) and AR19 (OSU-19) were obtained from Arno Therapeutics, Inc. (Parsippany, NJ). 4-Phenylbutyrate (4PBA) was obtained from Lancaster Synthesis Inc. (Ward Hill, MA). Trichostatin A (TSA), dimethyl sulfoxide (DMSO), methyl cellulose (molecular mass = 41,000 g/mol) and Tween-80 were obtained from Sigma-Aldrich (St. Louis, MO).

### Fibroblast cell culture

GM03813 fibroblasts^100^ (Coriell Cell Repositories; Camden, NJ) were derived from a type II SMA patient with deletion of both *SMN1* alleles and 3 copies of *SMN2*^101,102^. GM03814 fibroblasts^100^ (Coriell Cell Repositories) were derived from the carrier mother of GM03813 with 1 copy of *SMN1* and 5 copies of *SMN2*^101,102^. All fibroblast lines were maintained in Dulbecco’s modified essential medium (DMEM; Life Technologies, Grand Island, NY) containing 10% fetal bovine serum (EqualFetal; Atlas Biologicals, Fort Collins, CO), 2 mM L-glutamine (Life Technologies) and 1% penicillin/streptomycin (Life Technologies).

### Drug treatment of fibroblasts

For immunofluorescence, cells were seeded onto gelatinized glass coverslips at a density of 4000 cells/cm^2^. For mRNA expression analysis, fibroblasts were plated onto 6-well plates at a density of 3368 cells/cm^2^. Cells were plated onto 60-cm tissue culture-grade dishes at a density of 6667 cell/cm^2^ for protein analysis. Cells were treated with one of the following compounds (n = 3/dose): AR42 (10 nM–1 μM), AR19 (10 nM–1 μM), 4PBA (10 nM–1 μM), TSA (1–100 nM) or vehicle (DMSO). Test compounds were added to the medium at a 1:1000 dilution. Medium was changed daily for 5 days with fresh compound added daily.

### Immunofluorescence and gem count analysis

Immunostaining of fibroblast cells was accomplished as described previously^103,104^ using the mouse anti-SMN monoclonal antibody (mAb) (MANSMA2 (8F7); Developmental Studies Hybridoma Bank, Iowa City, IA^105^). SMN immunostaining within the nuclei of treated fibroblasts was visualized using a DMRXA2 epifluorescence microscope (Leica Microsystems Inc., Buffalo Grove, IL) with an ORCA-ER cooled camera (Hamamatsu, Hamamatsu City, Japan) and Volocity 6.1.1 software (Perkin-Elmer, Waltham, MA). For gem counting, the following parameters were measured in 100 randomly selected nuclei: the number of gems, the number of cells with gems and the number of cells with more than 1 gem.

### SMN2 promoter assay

The clone 11 cell line (Vertex Pharmaceuticals,^41^), which contains a β-lactamase reporter gene driven by the 3.4 kb *SMN2* promoter, was used in this assay. Cells were maintained in a humidified chamber at 37 °C and 5% CO_2_ with DMEM containing 5% EquaFETAL, 2 mM L-glutamine and 1% penicillin/streptomycin. They were seeded onto a black-walled, clear bottom 96-well tissue culture plates (Santa Cruz Biotechnology) at a density of 5 × 10^4^ cells/cm^2^. Drug compounds (n = 4/dose) were added to the medium using a 96-pin replicator (pin diameter = 1.19 mm; V&P Scientific, Inc., San Diego, CA) and plates were incubated for 19 h. 20 µL of 6X CCF2-AM dye (GeneBlazer In Vivo Detection Kit, Life Technologies) were added to each of the assay wells and plates were incubated at room temperature for 2 h before the plates are read on a fluorescence plate reader (λ_ex_ = 405 nm, λ_em_ = 530 nm and λ_em_ = 460 nm; Victor X4, Perkin Elmer). The 460 nm:530 nm fluorescence ratios were plotted against compound concentrations and used to generate a dose response curve for the *SMN2* promoter assay.

### Quantitative reverse transcriptase-polymerase chain reaction (qRT-PCR)

Total RNA was isolated from cell pellets or tissues treated with drug using RNeasy Mini columns (QIAGEN, Germantown, MD) according to manufacturer’s directions. cDNA was prepared from 0.5 to 1 μg total RNA using the iScript™ cDNA Synthesis Kit (Bio-Rad, Hercules, CA, USA) as per manufacturer’s instructions. Target transcripts were amplified via PCR from cDNA diluted 200–400-fold using the QuantiTect SYBR Green PCR kit (QIAGEN). The following primer sets (Integrated DNA Technologies; Coralville, IA) for the target transcripts were used: *full-length SMN* (*FL-SMN*) ^67^ (SMNex6F) 5′-ccatatgtccagattctcttgatga-3′, (SMNex78R) 5′-atgccagcatttctccttaattta-3′; *SMNΔ7*^67^ (SMNex6F), (SMNex68R) 5′-atgccagcatttccatataatagc-3′; *chondrolectin* (*Chodl*) (F) 5′-ttcatgggtctcttcaggttg-3′, (R) 5′-acctttaccagtggaatgacg-3′; *cyclin-dependent kinase inhibitor 1A* (*Cdkn1a*;^55^ (F) 5′-gacattcagagccacaggcacc-3′, (R) 5′-gagcgcatcgcaatcacggcgc-3′; *transmembrane protein 41b* (*Tmem41b*;^54^ (F) 5′-gaacgaaaagccttgtgcagaagc-3′, (R) 5′-ttcaccctctcttcctcactaagctg-3′; *Z*^+^ *agrin* (*Z*^+^*Agr*;^55^) (F) 5′-tgtcctgggggcttctctgg-3′, (R) 5′-gctgggatctcattggtcagctc-3′; *complement component 1 B polypeptide* (*C1qb*) (F) 5′-aggttttctccatgtgtcctg-3′, (R) 5′-ctctccaaactcaccaaggtc-3′; *AMPA-type ionotropic glutamate receptor 4* (*Gria4*) flip isoform^56^ (F) 5’-ggtgaatgtggacccaagga-3’, (R) 5′-gctacgttgctcaggctcaag-3′ and *Gria4* flop isoform^56^ (F) 5′-ggaggtgactccaaggacaaga-3′, (R) 5′-ccgccaaccagaatgtagaag-3′. Primers for the human reference transcripts *β-actin (ACTB*), *large ribosomal protein P0* (*RPLP0*) and *glyceraldehyde 3-phosphate dehydrogenase* (*GAPD*) as well as for the mouse reference transcripts *phosphoglycerate kinase* (*Pgk1*), *ribosomal protein L13a* (*Rpl13a*) and *β-glucuronidase* (*Gusb*) were purchased from Real Time Primers LLC (Elkins Park, PA). Quantitative PCR was performed in a 384 well plate on a 7900HT Fast Real-Time PCR system (Applied Biosystems, Foster City, CA). Each sample was assayed in triplicate.

The relative transcript levels were calculated using the efficiency-adjusted 2^−ΔΔCt^ method^106,107^. The PCR efficiency (E) for each primer set was calculated from the slope of a Ct versus log10(cDNA serial dilution) curve (E = 10^[−1/slope]^)^108^. ΔC_t,adjusted_ is the difference between the adjusted C_t_ (C_t,measured_ × E) for the target transcript and the geometric mean of the adjusted C_t_ values for the three reference genes^109^ and ΔΔC_t_ is defined as the difference between the ΔC_t_ for the SMA sample and the ΔC_t_ for the control sample. The reference transcripts were *ACTB*, *RPLP0* and *GAPD* for human cells and were *Pgk1*, *Rpl13a* and *Gusb* for mouse samples.

### Immunoblot

Fibroblasts treated with drug for 5 days or dissected spinal cords from animals treated with drugs for 5 days were homogenized in lysis buffer (0.1% Triton X-100 and Complete Protease Inhibitor cocktail (Roche Life Sciences, Indianapolis, IN) dissolved in PBS, pH 7.4). These samples were resolved through polyacrylamide gels containing 0.1% SDS via electrophoresis and transferred onto PVDF membranes via electroblotting as described previously^45^. For the detection of SMN protein, 100 μg of tissue protein extract was added to each lane of a midi-gel; 10 μg of protein extract were added to each lane of a mini-gel for all other proteins to be detected. Immunoblotting was completed as described in^45^. The following primary antibodies were used in this study: mouse anti-SMN mAb (1:500; MANSMA2,^105^ or 1:2000, clone 8, BD Biosciences), mouse anti-β-actin mAb (1:20,000; clone AC-15, Sigma-Aldrich), rabbit anti-histone H3 polyclonal antibody (pAb) (1:1000; Cell Signaling Technology, Beverly, MA), rabbit anti-acetyl-histone H3 (K9) mAb (1:1000; clone C5B11, Cell Signaling Technology), rabbit anti-Akt pAb (1:1000; Cell Signaling Technology), rabbit anti-phospho-Akt (S473) mAb (1:1000; clone 193H12, Cell Signaling Technology), rabbit anti-GSK3β mAb (1:1000; clone 27C10, Cell Signaling Technology), rabbit anti-phospho-GSK3β (S9) mAb (1:1000; clone 5B3, Cell Signaling Technology) and rabbit anti-GAPDH pAb (1:10,000; Sigma-Aldrich). The horseradish peroxidase-conjugated anti-mouse and anti-rabbit secondary antibodies (1:5000) were obtained from Rockland Immunochemicals, Inc. (Gilbertsville, PA). Band intensities were measured using ImageJ 1.45 s (National Institutes of Health, Bethesda, MD) or C-DiGit (Licor, Lincoln, NE). The full-length images for the immunoblots were provided in the Supplementary Information.

### Animals and ethical statement

SMNΔ7 SMA mice (*SMN2*^+*/*+^*; SMNΔ7*^+*/*+^*;mSmn*^*−/−*^)^13^ were generated from male and female carrier mice of the genotype *SMN2*^+*/*+^*; SMNΔ7*^+*/*+^*;mSmn*^+*/−*^ (line 4299; FVB.Cg-Tg(SMN2*delta7)4299Ahmb Tg(SMN2)89Ahmb Smn1^*tm1Msd*^; Jackson Laboratories, Bar Harbor, ME, #005025). As diet can affect the survival and phenotype of these mice^46^ as well as responsiveness to certain drugs^35,47^, the mice were fed the Harlan-Teklad 22/5 Rodent Diet for the experiments in this study. Neonatal offspring were genotyped using a PCR-based assay on genomic DNA from tail biopsies as described previously^13,44^. In these experiments, SMA mice are defined by the genotype *SMN2*^+*/*+^*;SMNΔ7*^+*/*+^*;mSmn*^*−/−*^ while nonSMA mice are either carrier (*SMN2*^+*/*+^*;SMNΔ7*^+*/*+^*;mSmn*^+*/−*^) or normal (*SMN2*^+*/*+^*;SMNΔ7*^+*/*+^*;mSmn*^+*/*+^) mice. Survival was defined by the following early removal criteria: greater than 20% loss of body mass within a period of 24 h, prolonged periods (greater than 10 min) of lethargy, aberrant observed respiration, non-responsiveness to tactile stimulation and loss of surface righting reflex after PND14. Those mice reaching these early removal criteria were ethically euthanized.

All animal experiments were conducted at the Ohio State University. They were conducted in accordance with the protocols described in the National Institutes of Health *Guide for the Care and Use of Animals* and were approved by the Ohio State University Institutional Laboratory Animal Care and Use Committee. These studies were completed in accordance with ARRIVE guidelines.

### Drug formulations and administration

AR42 and AR19 were dissolved in an aqueous solution containing 0.5% methyl cellulose and 0.1% Tween-80. All aqueous solutions were filter sterilized prior to injections. Beginning at either 4 or 9 days after birth (PND04 or PND09), SMNΔ7 SMA mice and their non-SMA littermates were treated with either AR42 (5 mg/kg/d), AR19 (5 mg/kg/d) or vehicle (0.5% methyl cellulose and 0.1% Tween-80 in ddH_2_O) by oral administration using a curved 18-gauge feeding needle (Harvard Apparatus, Holliston, MA) as described previously^110^. Drugs were administered to mice until the last SMA pup in the litter perished.

### Behavioral analysis

AR42-treated SMNΔ7 SMA mice were assessed for changes in vectorial movement duration, spontaneous locomotor activity and pivoting activity as described in^44^. All the measures were collected from treated mice at PND07, PND11 and PND14 using Stopwatch+ (Center for Behavioral Neuroscience, Atlanta, GA). Vectorial movement duration was measured as the amount of time each mouse was crawling (PND07) or walking (PND11 and PND14) within the viewing timeframe was collected. For spontaneous locomotor activity, each pup was placed in the center of a gridded (with 28 2.5-cm^2^ grids) arena and the number of grids crossed in 60 s was counted. For pivoting, each pup was placed in the center of a gridded arena and the number of times the pup turned 90 °C (pivots) during a 60-s time frame was counted. To minimize the stress on the pup, all motor phenotype assays were conducted in a single session.

### SMN enzyme-linked immunosorbent assay (ELISA)

Quantification of SMN protein levels in spinal cord extracts was measured using the SMN (human) Enzyme Immunometric Assay from Enzo Life Sciences (Farmingdale, NY) as described previously^111^ except that 40 μg of spinal cord extract were used for each sample. SMN concentrations were expressed as pg SMN per mL extract.

### snRNP assembly

In vitro snRNP assembly assays were performed with extracts from SMNΔ7 SMA mice treated with AR42 or vehicle for 5 days. Preparation of mouse tissue extracts and snRNP assembly experiments were carried out essentially as previously described^45,50^. The amount of immunoprecipitated U1 snRNAs was quantified using a STORM 860 Phosphorimager (GE Healthcare, Piscataway, NJ) and the ImageQuant version 4.2 software.

### HDAC fluorimetric assay

HDAC activity was measured using the Fluor de Lys fluorimetric assay (Enzo Life Sciences, Inc., Farmingdale, NY) according to manufacturer’s directions. SMNΔ7 SMA mice treated with AR42 or vehicle for 5 days beginning at PND04 and 25 μg of spinal cord protein extract were used for this assay. Fluorescence was measured using a SpectraFluor Plus fluorescence reader (TECAN, Morrisville, NC) using a λ_ex_ = 360 nm and a λ_em_ = 465 nm.

### Spinal cord histology

SMNΔ7 SMA mice and carrier littermates were treated with AR42 or vehicle beginning at PND04 until PND11. Treated mice were anesthetized with 2.5% Avertin and then transcardially perfused with ice-cold PBS followed by 4% paraformaldehyde in Sørensen’s phosphate buffer (100 mM Na_2_HPO_4_ and 100 mM NaH_2_PO_4_, pH 7.4). The lumbar spinal cords were postfixed with 4% paraformaldehyde in Sørensen’s phosphate buffer overnight at 4 °C followed by cryoprotection with 30% sucrose in ddH_2_O overnight at 4 °C. The lumbar spinal cords were sectioned transversely at a thickness of 25 μm using the MultiBrain^®^ Technology by NeuroScience Associates (Knoxville, TN). Every 12th section block was mounted onto a glass slide and stained with 1% Cresyl violet as described previously^36^. Motor neurons of the lumbar spinal cord—identified using the Allen Spinal Cord Atlas^112^ as a reference—in each 12th section were counted automatically using the Cell Counter (K De Vos, University of Sheffield) plugin of ImageJ^113^.

### Statistical analysis

Data were expressed as means ± standard error and were analyzed using one-way ANOVA with a Bonferonni post hoc test or unpaired t-tests. Kaplan–Meier analysis was performed on lifespan and onset of body mass loss data using the Mantel–Cox log rank post hoc test. Dose–response curves were generated using the four-parameter logistic formula. Goodness of fit was assessed with R^2^ and normality testing (Shapiro–Wilk). All statistical analyses were performed with SPSS v22.0 or SigmaPlot v12.

## Supplementary Information

Supplementary Information 1.

Supplementary Video 1.

## Data Availability

All data pertaining to this study are presented within the manuscript. Further inquiries related to data availability should be directed to M.E.R.B.
