# Supplementary material for: Evaluation of the orally bioavailable 4-phenylbutyrate-tethered trichostatin A analogue AR42 in models of spinal muscular atrophy
Source: Sci Rep. 2023 Jun 26;13:10374. doi: 10.1038/s41598-023-37496-0 (PMC10293174; doi:10.1038/s41598-023-37496-0)
Supplement: Supplementary file 1 — Supplementary Information 1. [file 41598_2023_37496_MOESM1_ESM.pdf]

Evaluation of the orally bioavailable 4-phenylbutyrate-tethered trichostatin A analogue AR42 in models of spinal muscular atrophy

Casey J. Lumpkin[1,2,#], Ashlee W. Harris[1,#], Andrew J. Connell[1,#], Ryan W. Kirk[1], Joshua A. Whiting[1], Luciano Saieva[3], Livio Pellizzoni[3,4,5], Arthur H. M. Burghes[6,7] and Matthew E. R. Butchbach[1,2,6,8*]

Supplementary Information

Supplementary Movie. Phenotype of SMNΔ7 SMA mice treated with AR42 at PND14. Spontaneous locomotor activity of SMNΔ7 SMA mice at PND14 that were treated with AR42 (5 mg/kg/day) or vehicle beginning at PND04. Age-matched, non-SMA littermates were also tested to serve as a healthy control.

Supplementary Data: Full-length Blot Images for Immunoblot Figures

Figure 3E

Probe:<br>SMN	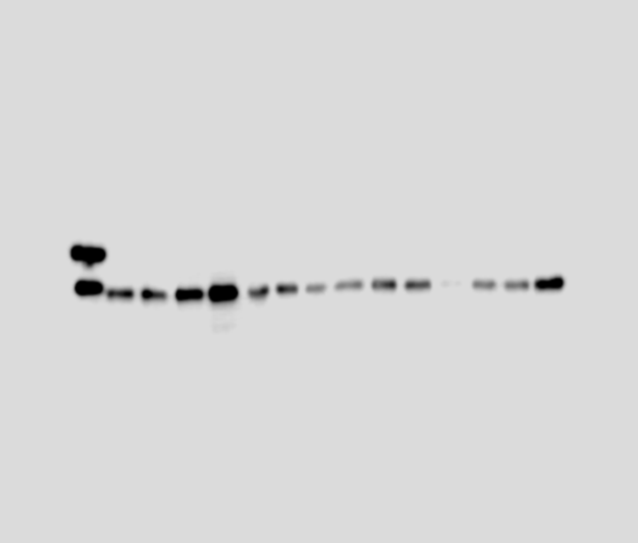
Probe:<br>β-actin	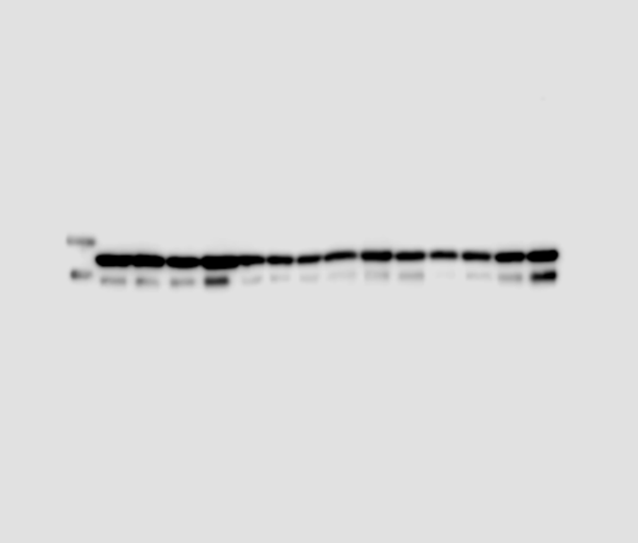

Figure 8B

Probes:<br>β-actin<br>SMN	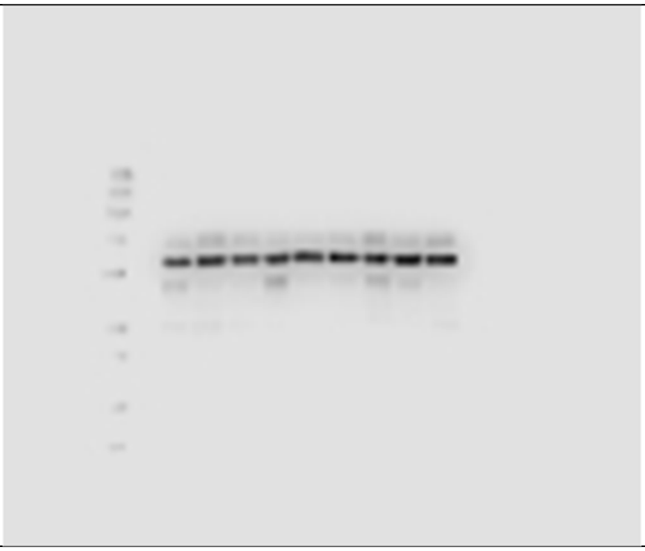

Figure 9B

Probe:<br>Ac-H3 (K9)	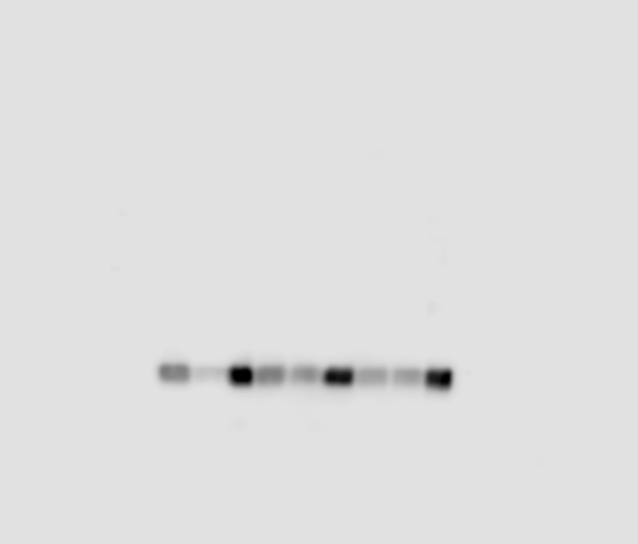

Probe: Total H3	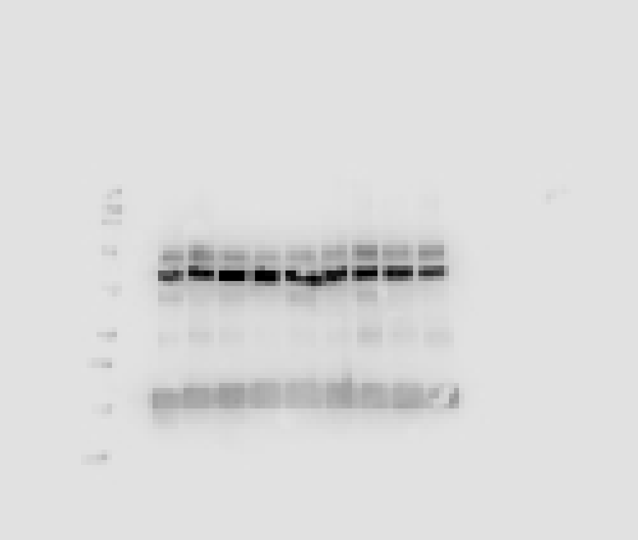
Probe: β-actin	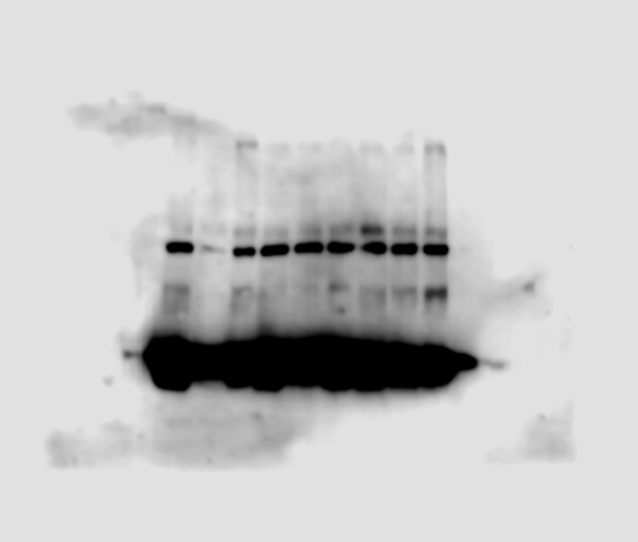

Figure 10A

Probe: P-AKT (S473)	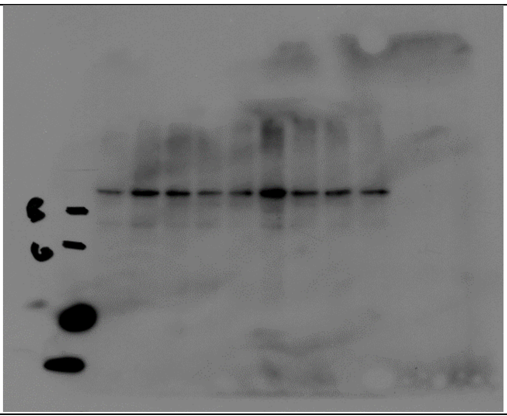

Probe: total AKT	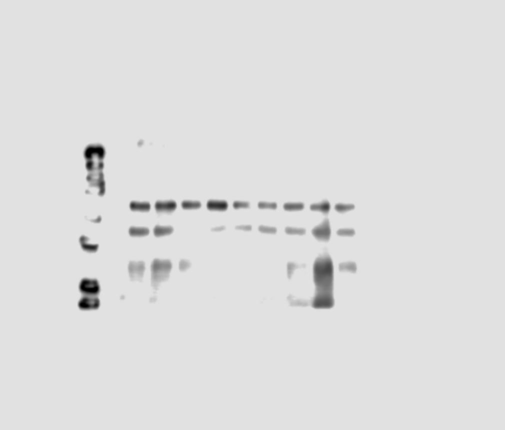
Probe: P-GSK3β (S9)	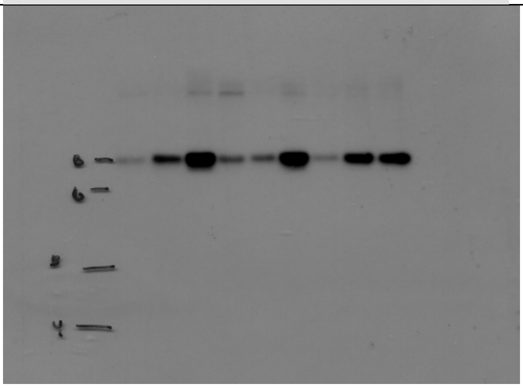
Probe: total GSK3	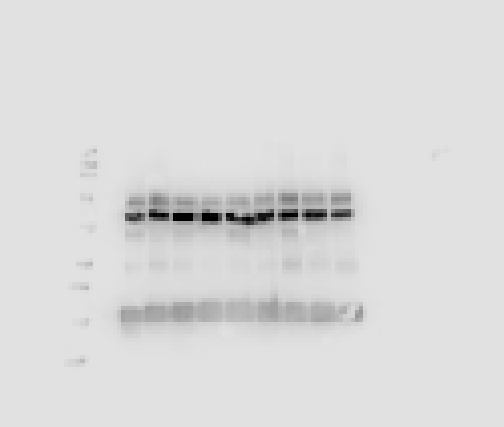
Probe: GAPDH	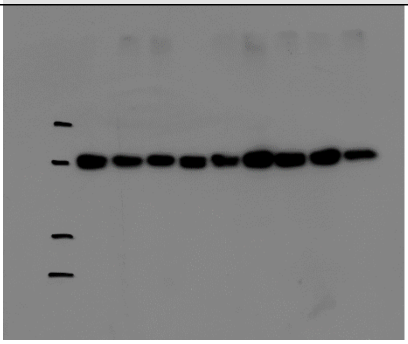
